# Identification of Multiple Hub Genes and Pathways in Hepatocellular Carcinoma: A Bioinformatics Analysis

**DOI:** 10.1155/2021/8849415

**Published:** 2021-07-12

**Authors:** Junwei Liu, Fang Han, Jianyi Ding, Xiaodong Liang, Jie Liu, Dongsheng Huang, Chengwu Zhang

**Affiliations:** ^1^Medical College of Soochow University, Suzhou 215006, China; ^2^General Surgery, Department of Hepatobiliary and Pancreatic Surgery and Minimal Invasive Surgery, Zhejiang Provincial People's Hospital, Affiliated People's Hospital, Hangzhou Medical College, Hangzhou, Zhejiang 310014, China; ^3^Hepatobiliary and Pancreatic Surgery Department, The Cancer Hospital of the University of Chinese Academy of Sciences (Zhejiang Cancer Hospital), Institute of Basic Medicine and Cancer (IBMC), Chinese Academy of Sciences, Hangzhou, Zhejiang 310022, China; ^4^Radiotherapy Department, Zhejiang Provincial People's Hospital, Hangzhou Medical College, Hangzhou, Zhejiang 310014, China

## Abstract

Hepatocellular carcinoma (HCC) is a common malignant tumor of the digestive system, and its early asymptomatic characteristic increases the difficulty of diagnosis and treatment. This study is aimed at obtaining some novel biomarkers with diagnostic and prognostic meaning and may find out potential therapeutic targets for HCC. We screen differentially expressed genes (DEGs) from the HCC gene expression profile GSE14520 using GEO2R. Gene Ontology (GO) analysis and Kyoto Encyclopedia of Genes and Genomes (KEGG) enrichment analysis were conducted by using the clusterProfiler software while a protein-protein interaction (PPI) network was performed based on the STRING database. Then, prognosis analysis of hub genes was conducted using The Cancer Genome Atlas (TCGA) database. Quantitative real-time polymerase chain reaction (qRT-PCR) was utilized to further verify the expression of hub genes and explore the correlation between gene expression and clinicopathological parameters. A total of 1053 DEGs were captured, containing 497 upregulated genes and 556 downregulated genes. GO and KEGG analysis indicated that the downregulated DEGs were mainly enriched in the fatty acid catabolic process while upregulated DEGs were primarily enriched in the cell cycle. Simultaneously, ten hub genes (CYP3A4, UGT1A6, AOX1, UGT1A4, UGT2B15, CDK1, CCNB1, MAD2L1, CCNB2, and CDC20) were identified by the PPI network. Five prognosis-related hub genes (CYP3A4, CDK1, CCNB1, MAD2L1, and CDC20) were uncovered by the survival analysis based on TCGA database. The ten hub genes were further validated by qRT-PCR using samples obtained from our hospital. The prognosis-related hub genes such as CYP3A4, CDK1, CCNB1, MAD2L1, and CDC20 could be considered potential diagnosis biomarkers and prognosis targets for HCC. We also use Oncomine for further verification, and we found CCNB1, CCNB2, CDK1, and CYP3A4 which were highly expressed in HCC. Meanwhile, CCNB1, CCNB2, and CDK1 are highly expressed in almost all cancer types, which may play an important role in cancer. Still, further functional study should be conducted to explore the underlying mechanism and biological effect in the near future.

## 1. Introduction

Hepatocellular carcinoma (HCC) is the most predominant primary liver cancer which ranks as the sixth most common neoplasm (4.7% of the total cases) and the fourth major cause of cancer mortality (8.2%) all over the world according to the GLOBOCAN 2018 report [1], and its incidence has been increasing in the recent decades [2]. Despite numerous advancements into the treatment innovation of pharmacotherapy and operative treatment and even interventional therapy, the overall survival of HCC still remains unsatisfactory with little improvement in the last decade because of the high recurrence rate and intra- or extrahepatic metastasis [3]. Regretfully, HCC is generally diagnosed at advanced stages or with distant metastasis owing to deficiency of early clinical symptoms and unelucidated pathogenesis, which increases the difficulty of treatment and leads to an unfavorable prognosis [4]. Therefore, for early detection and better prognosis of HCC, it is urgently needed to identify a novel biomarker with diagnostic and prognostic significance.

With the coming of the age of big data, bioinformatics has attracted widespread attention and gradually plays an essential role in biomedical research and disease mechanism exploration. According to recent bioinformatics analysis, the MAPK and IGF1R signal pathway was related to obesity [5]. In particular in microarray analysis based on high-throughput platforms, it is an invaluable and powerful method to screen many differentially expressed genes (DEGs) associated with tumorigenesis and progression from gene expression profiles [6, 7]. Seven core genes are considered to be targets for developing therapeutics against both familial hypercholesterolemia and atherosclerosis [8]. Network analysis of transcriptomics data was used for the prediction and prioritization of idiopathic pulmonary fibrosis (IPF). Seven genes were related to IPF, and most pathways were membrane transport and signal transduction [9]. The systemic lupus erythematosus (SLE) dataset GSE30153 was conducted for further analysis. Four genes were found to be associated with SLE. And dysregulated pathways might be associated with SLE development and progression [10]. However, every newly emerging thing has their superiority and limitation. There is no doubt that the development of bioinformatics and establishment of open databases assist researchers in easily accessing abundant data from various resource banks and go a step further [11, 12]. But it has gradually been realized that only datasets with relatively large sample sizes or multiple microarray datasets and integrated bioinformatics approaches are able to decrease bias and inaccurate results caused by limited sample size and heterogeneity of a single cohort [13, 14]. What is more, clinical sample validation is a crucial step to improve the predictive power and accuracy. For example, expression profiles GSE6477 and GSE47552 were used, and 51 upregulated and 78 downregulated DEGs were identified. Survival analysis was evaluated to verify key hub genes that could affect the prognosis of multiple myeloma [15]. In addition, 10 hub genes and four core genes were strongly linked to ovarian cancer, for which we could provide potential molecular biomarkers for diagnosis and treatment targets in the future [16]. Bioinformatics is used to discover single-target specialized research. For example, ADAMTS9-AS1 influences prostate cancer cell proliferation, and it functioned as ceRNA, effectively becoming a sponge for hsa-mir-96 and modulating the expression of PRDM16 [17]. Other researchers construct an immune signature model based on seven immune-related genes in the recognition of disease progression and prognosis of lung squamous cell carcinoma patients [18].

**Figure 1:**
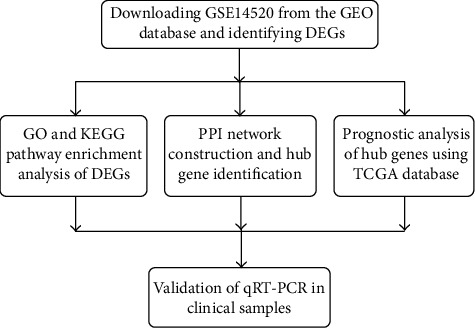
Flow process diagram of present study. GEO: Gene Expression Omnibus; DEG: differentially expressed gene; GO: Gene Ontology; KEGG: Kyoto Encyclopedia of Genes and Genomes; PPI: protein-protein interaction; TCGA: The Cancer Genome Atlas; RT-qPCR: quantitative real-time polymerase chain reaction.

In the present study, we made use of bioinformatics analyses to screen DEGs from Gene Expression Omnibus (GEO). Afterwards, Gene Ontology (GO) analysis, Kyoto Encyclopedia of Genes and Genomes (KEGG) enrichment analysis, and a protein-protein interaction (PPI) network were employed to obtain the in-depth understanding of the possible functions of the DEGs and find out hub genes, respectively. Then, prognosis analysis of hub genes was conducted using The Cancer Genome Atlas (TCGA) database. At last, reverse transcription quantitative real-time polymerase chain reaction (RT-qPCR) was utilized to further verify the expression of hub genes and explore the correlation between gene expression and clinicopathological parameters. In brief, the aim of this research is to provide valuable clues for pathogenesis mechanism elucidation of HCC and obtain some novel biomarkers with diagnostic and prognostic meaning for HCC. A flow process diagram presenting the experimental design is displayed in Figure 1.

## 2. Materials and Methods

### 2.1. Data Acquisition

We downloaded the gene expression profile of GSE14520 from the GEO datasets, an international public and free repository for researchers to easily access the raw data, processed data, or metadata (https://www.ncbi.nlm.nih.gov/gds/) [19]. 220 normal liver tissues and 225 HCC tissues were included in GSE14520, which was constructed based on the GPL3921 platform ([HT_HG-U133A] Affymetrix HT Human Genome U133A Array) and GPL 571 platform ([HG-U133A_2] Affymetrix Human GeCnome U133A 2.0 Array) from Jan 22, 2009.

### 2.2. Identification of DEGs

The raw data of the gene expression profile submitted by original authors was analyzed by R version 3.5.1. In the present study, we apply GEO2R (https://www.ncbi.nlm.nih.gov/geo/geo2r/), an online interactive analysis tool, which allows users to analyze the degree of data discrepancy between different groups in GEO to screen the DEGs between cancerous and noncancerous samples. To decrease the false positive rate, *p* values were adjusted in accordance with the Benjamini-Hochberg false discovery rate (FDR) method. And FDR < 0.05 and ∣logFC | >1 were set as the criteria to screen out DEGs.

### 2.3. GO Functional and KEGG Pathway Analysis of DEGs

**Figure 2:**
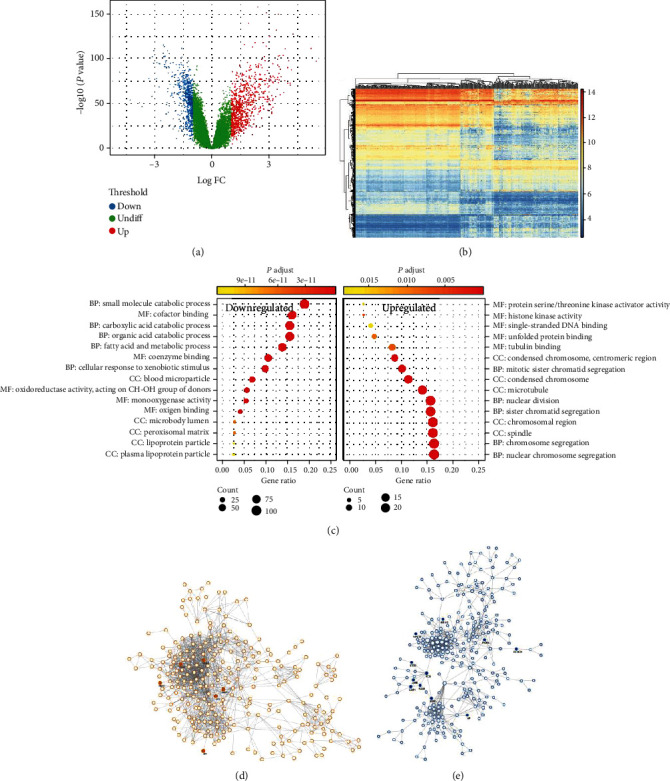
Various results of bioinformatics analyses in DEGs and hub genes. (a) Volcano plot of the genome-wide detected in GSE14520. Red: upregulated; green: no difference; blue: downregulated. FDR < 0.05 and ∣logFC | >1 were set as the threshold. (b) Heatmap of 1053 DEGs between HCC samples and normal tissues in GSE14520. The deeper the color, the more greatly the relative expression of DEGs goes up or down. Red: upregulated; blue: downregulated. (c) GO analysis of the down- and upregulated DEGs in HCC. The *y*-axis presents significantly enriched GO annotation terms, and the *x*-axis presents the different gene ratios. (d) The PPI networks of upregulated DEGs in HCC. (e) The PPI networks of downregulated DEGs in HCC. The orange nodes represent upregulated DEGs while the blue ones represent downregulated DEGs. The sizes of nodes mean the score levels of DEGs. A larger node represents a higher score. Solid line between two nodes represents the interaction between two DEGs. DEGs: differentially expressed genes; FDR: false discovery rate; FC: fold change; HCC: hepatocellular carcinoma; GO: Gene Ontology; PPI: protein-protein interaction.

GO analysis is a predominant bioinformatics tool for annotations of genes and their products, including three categories: cellular components (CC), molecular function (MF), and biological pathways (BP) [20]. KEGG is an aggregation of databases which consist of information about genomes, biological pathways, diseases, and chemicals [21]. The clusterProfiler package was employed to perform GO functional enrichment analysis and KEGG pathway analysis for DEGs in R studio version 1.1.456. The adjusted *p* < 0.05 was regarded as statistically significantly different (Figure 2(c)).

### 2.4. PPI Network Analysis and Identification of Hub Genes

The interactional correlation of DEGs was assessed by the Search Tool for the Retrieval of Interacting Genes (STRING) online database (https://string-db.org), which contains a great quantity of known and predicted protein-protein interactions in organisms [22]. We defined the confidence score > 0.9 as the cut-off criterion for the interaction between the two proteins. Cytoscape, software for visualization of the topological network, was applied to visualize the PPI network. Meanwhile, a scoring analysis of the protein network was conducted by CentiScaPe 2.1, a plug-in component of Cytoscape. Subsequently, proteins with top ten scores were selected out as the hub genes in the network.

### 2.5. Survival Analysis of Hub Genes from TCGA Database

There are comprehensive multidimensional atlases of key genomic changes in various types of cancer in TCGA (https://cancergenome.nih.gov/), which is a joint effort between the National Cancer Institute (NCI) and National Human Genome Research Institute (NHGRI) [23]. In this study, we analyzed the prognosis value of hub genes using a dataset of 364 case samples from TCGA database. Kaplan-Meier curves for high- and low-expression groups were drawn by the median value of each hub gene using the logrank method.

### 2.6. Patient Samples

A total of 59 pairs of HCC samples and adjacent noncancerous tissues (distance from tumor > 5 cm) were obtained from the Department of Hepatobiliary and Pancreatic Surgery and Minimally Invasive Surgery of Zhejiang Provincial People's Hospital (Hangzhou, China). It consists of 46 males and 13 females aged 30-77 (58.80 ± 10.99) years. None of the above patients received preoperation radiotherapy or chemotherapy. The study has been conducted with the World Medical Association Declaration of Helsinki. Meanwhile, the protocol of the study was approved by the Ethics Committee of Zhejiang Provincial People's Hospital (no. 2020QT043). And the informed consent had been obtained from all included patients prior to this research. In addition, the HCC samples and paired adjacent noncancerous tissues were promptly frozen in liquid nitrogen after removal from patients and stored at -80°C. All the tissue specimens were pathologically confirmed as HCC, and the clinical stage of the tumor was determined according to the Cancer Staging Manual of the American Joint Committee on Cancer (version 8, 2017) while the tumor pathological differentiation stage was defined by the Edmondson-Steiner classification. The follow-up ended in October 2019 or at death.

### 2.7. RT-qPCR Validation Analysis

The specific protocols are as follows: first, total RNA of matched samples was extracted at 4°C temperature using the TRIzol reagent (Life Technologies, USA); second, the PrimeScript™ RT reagent kit (Takara, Japan) was used for reverse transcription of RNA into complementary DNA. Last, the RT-qPCR was carried out on a ABI ViiA 7 Real Time PCR System (Thermo Fisher, USA) with a SuperReal SYBR Green Premix Plus (Tiangen Biotech, China) as a fluorescent dye. GAPDH was chosen as the internal reference in the present study. All the experiments were in triplicate independently. All the primers of each hub gene are shown in Table 1. The 2^−ΔΔCt^ method was employed to evaluate the relative expression of each hub gene [24].

### 2.8. Oncomine Verification

In order to further verify the results, we used the Oncomine database (http://www.oncomine.org) to screen hub genes which were AOX1, CCNB1, CCNB2, CDK1, UGT1A4, and UGT2B15. The Oncomine database is a comprehensive tumor gene expression dataset. It mainly summarizes the tumor sequencing data of the GEO and ArrayExpress. In the Oncomine database, we can check the expression of multiple genes in multiple tumors. We tested the dataset of six differential genes in the dataset of Oncomine.

### 2.9. Statistical Analysis

In the present study, the SPSS 25.0 statistical software package was applied for statistical analysis. All continuous variables were expressed as mean ± standard deviation (SD). The hub gene expression which was calculated by 2^−ΔΔCt^ methods was further processed by the log_2_ transform. Student's two-tailed *t*-test was utilized to explore the association between gene expression and clinicopathological features of HCC patients. Graphpad Prism 8.0 was also applied to draw a diagram of pairing samples. *p* < 0.05 was considered statistically significant.

## 3. Results

### 3.1. Identification of DEGs in HCC

GSE14520, which contains 225 HCC tissues and 220 normal tissues, is a large-sample gene expression microarray. In the present study, we obtained the abovementioned gene expression profile from GEO which was further analyzed. The result showed that a total of 1053 DEGs were captured, 497 of which were upregulated genes while 556 of which were downregulated. A volcano plot was plotted by GEO2R to visualize the distribution of DEGs (Figure 2(a)), using the false discovery rate (FDR) < 0.05 and ∣logFC | >1 as screening criteria. For better differentiation, significantly upregulated or downregulated genes were shown by red or blue dots, respectively. Moreover, a heatmap was also generated by GEO2R to exhibit the relative expression levels of DEGs in GSE14520 (Figure 2(b)). Rows and columns represent different DEGs and independent samples, respectively. Also, in the volcano plot, red and blue separately represent up- and downregulated DEGs. And the deeper the color, the more greatly the relative expression of DEGs goes up or down.

### 3.2. GO Analysis and KEGG Enrichment Pathways of DEGs

For a more comprehensive understanding to the functional characteristics, we applied GO and KEGG analysis to DEGs using the clusterProfiler package. On the one hand, GO analysis results showed that significant differences were uncovered in 823 terms for downregulated genes while 499 terms for upregulated genes. As illustrated in Figure 2(c), for downregulated DEGs, genes were mainly enriched in “small molecule catabolic process” and “acid catabolic process (including organic acid and fatty acid)” in terms of BP. Regarding MF, the abovementioned DEGs were particularly enriched in “cofactor binding.” As for the CC group, the genes were strongly enriched in “blood microparticle.” For upregulated DEGs, “chromosome segregation” has the highest enrichment of BP. With regard to the MF group, the main enrichment functions included “tubulin binding,” “unfolded protein binding,” and “single-stranded DNA binding.” And in the CC classification, the genes were dominantly enriched in the following components: “spindle,” “chromosomal region,” and “microtubule.” On the other hand, KEGG analysis uncovered that the pathways enriched by 368 downregulated DEGs were strongly associated with “chemical carcinogenesis,” “fatty acid degradation,” “drug metabolism,” “bile secretion,” and “peroxisome proliferator-activated receptor (PPAR) signaling pathway” while 83 upregulated DEGs were obviously enriched in “cell cycle,” “DNA replication,” and “pyrimidine metabolism” (Table 2). The above results of two analyses concluded that these DEGs had a tight association with cancer-related metabolism and cell proliferation and might modulate these two processes through multiple pathways.

### 3.3. PPI Network Establishment

Based on the STRING database, the PPI network with upregulated DEGs or downregulated DEGs was visualized by the Cytoscape software, respectively. In terms of upregulated DEGs, the network contained 496 nodes and 1515 edges with an average node of 6.11 (Figure 2(d)). As for downregulated DEGs, the network consisted of 544 nodes and 885 interactions with an average node of 3.25 (Figure 2(e)). Interestingly, in both up- and downregulated gene networks, the *p* value of PPI enrichment was <1 × 10^−16^. Furthermore, we employed Cytoscape to filter out proteins with top five scores as hub genes, separately. The detailed information of ten hub genes with the highest score is displayed in Table 1.

### 3.4. Prognosis Analysis of Hub Genes in HCC

**Figure 3:**
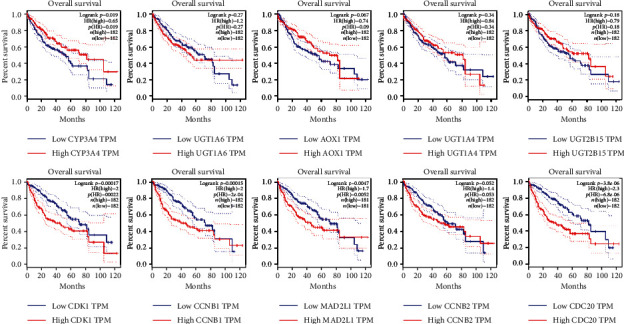
Survival curves of ten hub genes in hepatocellular carcinoma.

We obtained a total of 364 cases of correlative HCC clinical data from TCGA database to further explore the prognosis value of each hub gene by drawing the Kaplan-Meier curves. HCC patients were divided into two groups depending on the expression level of each hub gene. The results uncovered that only a half of hub genes were tightly associated with the poor prognosis of HCC, including CYP3A4, CDK1, CCNB1, MAD2L1, and CDC20 (Figure 3). Moreover, only CYP3A4 was the downregulated gene among abovementioned prognosis-related hub genes while the others were upregulated genes.

### 3.5. RT-qPCR Validation of Expression of Ten Hub Genes in HCC Clinical Samples

**Figure 4:**
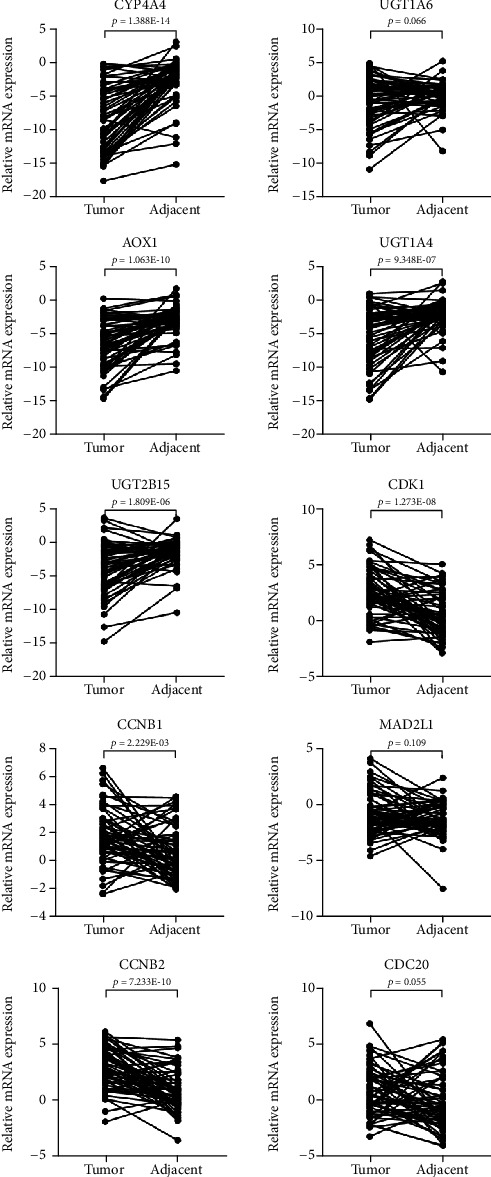
qRT-PCR validation of mRNA expression levels of ten hub genes in paired HCC samples. The *x*-axis represents different groups, and the *y*-axis represents the relative expression of genes. Expression data of each hub gene was processed by the 2^−ΔΔCt^ method and log2 transform. The statistical significance was evaluated using the paired *t*-test. qRT-PCR: quantitative real-time polymerase chain reaction; HCC: hepatocellular carcinoma; ns: no significance.

To further validate the aforementioned bioinformatics analysis, the mRNA expression levels of these hub genes were obtained by the RT-qPCR experiment in 59 pairs of HCC and adjacent noncancerous tissues. As suggested in Figure 4, there were significant differences between HCC samples and adjacent noncancerous tissues in seven hub genes, including CYP3A4, AOX1, UGT1A4, UGT2B15, CDK1, CCNB1, and CCNB2. Interestingly, each of ten genes had the upregulation or downregulation tendency in HCC as previously predicted by other microarray and network analyses.

Besides, we explored the connection between clinicopathological parameters and expression levels of ten hub genes (Supplementary Table S1). The unequal total number of cases in each group is due to data missing in mRNA expression or clinicopathological parameters of some samples. Statistical analysis suggested that there were significant associations between the expression of quite a few hub genes and variables such as histological differentiation, satellite lesions, pN, cirrhosis, and serum albumin. Speaking concretely, the relative expression of CYP3A4 was notably associated with histological differentiation and pN. The relative expression of UGT1A6 was notably associated with cirrhosis and serum AFP. And AOX1's expression was remarkably related to histological differentiation, satellite lesions, and vascular cancer embolus while UGT1A4's expression was remarkably related to histological differentiation, satellite lesions, pN, and cirrhosis. For UGT2B15, connections between expression and parameters including tumor necrosis, satellite lesions, and pN were significant. For CDK1, connections between expression and parameters including histological differentiation and serum CEA were significant. Cirrhosis and serum albumin were proven to have a tight relation with CCNB1's expression. The relative expression of CCNB2 was tightly associated with serum albumin. At last, CDC20's expression was remarkably related to serum AFP and albumin. Except for MAD2L1, each hub gene had one or more than one parameter associated with its expression level, which confirmed that the majority of hub genes might become a novel biomarker of HCC potentially.

### 3.6. Oncomine Validation of Expression of Six Hub Genes in Multicancer

**Figure 5:**
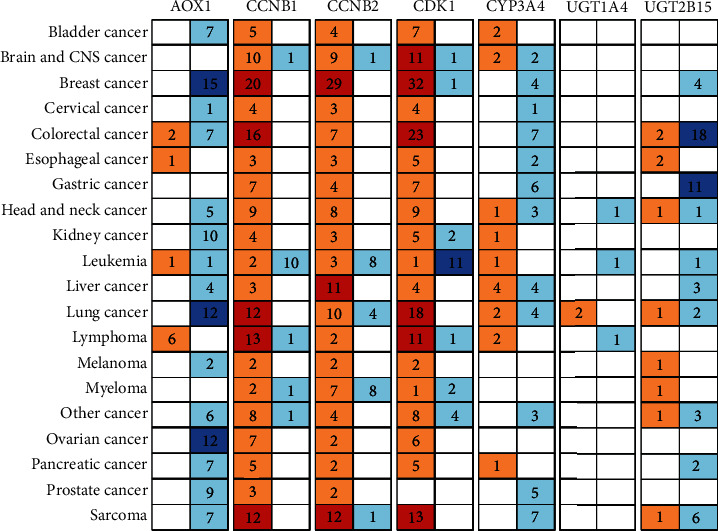
Oncomine verification results. Every two columns in the figure are a gene. The first column of each gene represents whether it is highly expressed in tumors. The second column represents whether it is highly expressed in normal tissues. The numbers in the figure represent the number of datasets that specifically support high and low expression conclusions. The darker the color, the more datasets are supported.

Six genes of our own samples were found to have expression difference by qPCR in hepatocellular carcinoma. To ensure that these core genes are really meaningful, we used the Oncomine database for further verification of differential genes which are CDK1, AOX1, CCNB1, CCNB2, CYP3A4, and UGT2B15. After comparing all datasets contained in Oncomine, we found that there are four genes that are different in HCC (Figure 5). They are CCNB1, CCNB2, CDK1, and CYP3A4. It is worth noting that CCNB1, CCNB2, and CDK1 are highly expressed in almost all kinds of tumors, so it is possible that these three genes are essential for the occurrence of tumors.

## 4. Discussion

Despite the great improvements on treatment approaches for HCC in recent decades, such as from laparotomy liver resection to laparoscopic hepatectomy or even liver transplantation, radiofrequency ablation to transcatheter arterial chemoembolization, and sorafenib to programmed cell death-1, they had little effect to hinder the increasing mortality year by year [25]. In another word, to obtain a better prognosis of HCC, regular surveillance and early diagnosis might be the critical principles [26]. In recent years, with the rapid development of various bioinformatics databases and high-throughput researches, researchers could mix multiple bioinformatics methods to deeply explore the crucial pathogenesis and clinical diagnosis or prognosis of different diseases from the molecular plane [27]. For instance, Li et al. [12] uncovered that TOP2A, CCNB1, and KIF4A might promote the development of HCC, especially in proliferation and differentiation. Furthermore, Zhou et al. [28] also screened 15 hub genes and pathways to identify potential prognostic markers for HCC treatment by integrated bioinformatics analysis. Regretfully, in spite of more and more discovery of candidate biomarkers for HCC, there still are a portion of patients who are unable to obtain early diagnosis and prognosis prediction. Therefore, more reliable and credible studies with validation in vivo and in vitro are preferred in the future so that the conclusions of higher-level evidence could be put into clinical practice or conducted in clinical work.

The present study downloaded the GSE14520 microarray which consists of 225 HCC samples and 220 nontumor samples from the GEO database. Its relatively large sample size and long time span could also reduce the correlative biases. 497 upregulated DEGs and 556 downregulated DEGs were picked out by gene expression analysis of the whole genome conducted on the abovementioned gene expression profile. Subsequently, the results of GO analysis determined that downregulated DEGs were associated with the fatty acid catabolic process in biological pathways. As known to all, the liver is a vital organ in the human body which is major in metabolism. When HCC occurs, with the change of the internal environment and biological function of the liver, fatty acid is immoderately used by cancer cells as cellular building blocks to generate membrane structures and product signaling molecules, which further leads to dysregulated fatty acid metabolism [29, 30]. Interestingly, fatty acid degradation was also significant in KEGG pathway analysis of downregulated DEGs, which was in accordance with the results of GO. What is more, the downregulated DEGs had a tight relationship with PPAR signaling pathway as well according to the KEGG analysis. Early in 2009, Cao et al. had already found that HCC could be sensitized to 5-fluorouracil antitumor activity through the activation of PPAR gamma signaling pathway, which meant PPAR signaling pathway played pivotal roles in anticancer effect to HCC [31]. With regard to upregulated DEGs, both GO and KEGG analyses indicated that it was notably related to cell division and cell cycle. It seems like reasonable because frequent cell proliferation and accelerated cell cycle are both key points in tumorigenesis of HCC.

Ten hub genes were obtained by PPI, comprising CYP3A4, UGT1A6, AOX1, UGT1A4, UGT2B15, CDK1, CCNB1, MAD2L1, CCNB2 and CDC20. The former five were downregulated genes and the latter five were upregulated ones. More importantly, five hub genes were validated to have a notable connection with prognosis based on TCGA database. Only CYP3A4 is the downregulated gene among the five prognosis-related hub genes, and another four genes (CDK1, CCNB1, MAD2L1, and CDC20) were related to mitosis in the light of results of pathway enrichment analyses. Among these five hub genes associated with HCC overall survival, a good number of studies have revealed their essential role in tumor. CDK1, which is a member of Ser/Thr protein kinase family, encodes cyclin-dependent kinase 1. And the latter plays an essential role in cell cycle G2/M transition, which was verified by Wang et al. [32] and Gao et al. [33]. The protein encoded by CCNB1 was a kind of regulatory protein involved in mitosis. Gu et al. illustrated in his study that CCNB1 was an upregulated and prognosis related gene in HCC using TCGA cohort [34]. MAD2L1 is an integral part of the mitotic spindle assembly checkpoint, which ensures that all chromosomes are correctly aligned on the metaphase plate. And it was verified that increased expression of MAD2L1 might be a biomarker for diagnosis and prognosis in patients with HCC [35]. CDC20 encodes a regulatory protein which interacts with the anaphase-promoting complex/cyclosome in the cell cycle. Li et al. [36] obtained CDC20 gene by molecular interaction networks and further confirmed the high expression level in HCC tumors by RT-qPCR, western blot and immunohistochemistry. CYP3A4 is 3A4 isoform of cytochrome P450 superfamily. Its encoding protein is a kind of monooxygenase located in the endoplasmic reticulum, which catalyzes a good number of reactions involving drug metabolism and lipid synthesis. And glucocorticoid and some pharmacological reagents could induce its expression [37]. Multivariate analysis uncovered downregulation of the CYP3A4 gene as an independent predictor for overall survival and early recurrence [38]. Based on existing literature, it is not difficult to discover that all of upregulated genes (CDK1, CCNB1, MAD2L1, and CDC20) are involved in the regulation of cell cycle, while the downregulated gene CYP3A4 is mainly related to the metabolism of diverse drugs and various lipid reactions in vivo, which are consistent with the results of path enrichment analysis.

GSE14520 contained clinical samples with 225 HCC samples and 220 nontumor samples. This is a relative large scale dataset. Some of researchers have used this cohort studied by their view of immune environment in primary cancer. Li et al. have validated the GSE14520 and found that there were multifunction-related subtypes which could affect different immune and clinical characteristics [39]. Sun et al. have found 33 immune gene pairs which could establish the immune-related signature of prognosis. They found a link between immune microenvironment and prognosis, which could be a promising predictor for HCC patients [40]. These two studies were promising for immune related prognosis prediction of HCC. Our study were focus on the clinical samples for prognosis not only immune related, but for potential diagnosis biomarkers and prognosis targets with external verification. Other researchers have verified fourteen genes related to cell signaling pathways which could be used to predict HCC recurrence [41]. The idea of this research is great using GSE for external verification. Also presented in another studies, Liu et al. have found a novel robust four-gene metabolic signature for HCC prognosis prediction [42]. Ouyang et al. identified a 12 hub gene-related DNA methylation-driven genes which could be a risk and prognosis factor for HCC patients [43]. Nomogram was included in these two studies with the signature of some characteristics for overall survival prediction. However, we performed not only the hub genes which could lead to poor prognosis but also the cell signal pathway for tumorigenesis. Further, we verified our conclusion through external verification of patient tissue samples. The cohort of the expression profile GES14520 was also used to discover more specific topics, such as UBE2C for the therapeutic target [44], a ceRNA network as a biomarker for prognosis [45], and ten exosome-related hub genes as a target for treatment [46]. All these researches partially or fully used the cohort of GES14520. Furthermore, Liao et al. have studied the diagnostic and prognostic values of minichromosome maintenance (MCM) gene expression [47]. Ding et al. confirmed that downregulation of AGXT2L1 promotes the lipogenesis of HCC cells. These two findings also revealed some internal mechanism through GES14520. We analyzed GES14520 to verify the signal pathways thoroughly and further verified the hub genes by external verification. According to these surveys, not only does this prove that the results are authentic and credible by GES14520 but also there are many different aspects that have not been fully discovered. At last, we further confirmed six hub genes by the Oncomine database. As Oncomine contains GEO and ArrayExpress datasets, we finally used the Oncomine database to reverify the expression of six hub genes in HCC. We found that four genes are highly expressed in HCC, among which four genes, CCNB1, CNB2, CDK1, and CYP3A4, are highly expressed in tumors, while CCNB1, CCNB2, and CDK1 are highly expressed in almost all cancer types, which may be involved in the important tumorigenesis or progression as mentioned above.

Although integrated bioinformatics analysis and clinical sample validation were performed in the present study, there were still some limitations: first, only one gene expression profile GSE14520 was mined from the GEO database. It may lead to less reliable and accurate results in differential gene analysis compared with the multiple microarray study in spite of the large size sample in GSE14520. Secondly, compared with GSE14520, the important aspects of clinical samples recruited in this study, such as histological type, tissue location, and clinical information, are almost the same. Although there is selection bias, such as race and geography, the prediction results are still credible by external verification. Besides, due to uneven global population distribution and unequal prevalence of the exposure risk factors, most HCC cases (80%) are concentrated in sub-Saharan Africa and Eastern Asia [2], and this is why we chose all cases from Southeast Asia to ensure the academic value of this study. Thirdly, a more comprehensive study contains the underlying mechanism, and the biological effect should be further conducted in the near future. At last, we only used 58 paired samples for qPCR verification, but we still need to verify the proteomics conclusions in the near future.

In conclusion, the tumorigenesis of HCC is a multigene disease. This study employed a bioinformatics analysis by using GSE14520 to analyze the DEGs. The aim of this study is to provide a basis for in-depth understanding between HCC and DEGs. In addition, the prognosis-related hub genes such as CYP3A4, CDK1, CCNB1, MAD2L1, and CDC20 could be considered potential diagnosis and prognosis biomarkers for HCC. Furthermore, we considered that multicell signal pathways might affect the tumor progression which could be the therapeutic and diagnosis targets for HCC. Although the external verification is included in this study, further functional study is still needed to validate the role in DEGs and HCC.

## Figures and Tables

**Table 1:** The detailed information and primer sequences of ten hub genes screened out by PPI networks.

Category	Gene abbreviation	Description	Forward primer (5′-3′)	Reverse primer (5′-3′)
Upregulated	CDK1	Cyclin-dependent kinase 1	CAGGTCAAGTGGTAGCCATG	ACCTGGAATCCTGCATAAGC
	CCNB1	Cyclin B1	AAGGCGAAGATCAACATGGC	CCAATGTCCCCAAGAGCTGT
	MAD2L1	Mitotic arrest deficient 2 like 1	CGGTGACATTTCTGCCACTG	GGTCCCGACTCTTCCCATTT
	CCNB2	Cyclin B2	CTGTACATGTGCGTTGGCAT	CTTGGAAGCCAAGAGCAGAG
	CDC20	Cell division cycle 20	CAGCAGAAACGGCTTCGAAA	ACCCGAACATCATGGTGGTG

Downregulated	CYP3A4	Cytochrome P450 3A4	TGAAAGAAAGTCGCCTCGAA	CCAGATCGGACAGAGCTTTG
	UGT1A6	Uridine diphosphate glucuronosyl transferase 1A6	CCGTGTTCCCTGGAGCATAC	AGGAAGTTGGCCACTCGTTG
	AOX1	Alcohol oxidase 1	AATTCCTCAGCAAGTGCCCT	CGGAAGGCTGACACAAATTC
	UGT1A4	Uridine diphosphate glucuronosyl transferase 1A4	TGCCATACTTTTTCTGCCCC	AACAGCCACACGGATGCATA
	UGT2B15	Uridine diphosphate glucuronosyl transferase 2B15	CTGGAAGCTGTGGAAAGGTG	CACCTCATGACCCCTCTGAA

PPI: protein-protein interaction.

**Table 2:** KEGG analysis of the down- and upregulated DEGs in HCC.

Category	ID	Description	Gene ratio	*p* value	*p*.adjust
Downregulated	hsa05204	Chemical carcinogenesis	27/368	6.38*E* − 16	1.70*E* − 13
	hsa00071	Fatty acid degradation	19/368	4.55*E* − 14	3.02*E* − 12
	hsa00982	Drug metabolism-cytochrome P450	23/368	2.03*E* − 13	1.08*E* − 11
	hsa04976	Bile secretion	19/368	8.13*E* − 10	2.16*E* − 08
	hsa03320	PPAR signaling pathway	19/368	1.06*E* − 09	2.55*E* − 08

Upregulated	hsa04110	Cell cycle	11/83	1.04*E* − 07	2.03*E* − 05
	hsa03030	DNA replication	6/83	2.41*E* − 06	0.000234
	hsa00240	Pyrimidine metabolism	7/83	0.000124	0.008017

KEGG: Kyoto Encyclopedia of Genes and Genomes; DEGs: differentially expressed genes; HCC: hepatocellular carcinoma.

## Data Availability

All data generated or analyzed during this study are included in this article.

## Supplementary Materials

Supplementary Materials See Table S1 for the connection between clinicopathological parameters and expression levels of ten hub genes.
