# Supplementary material for: Identification of Multiple Hub Genes and Pathways in Hepatocellular Carcinoma: A Bioinformatics Analysis
Source: Biomed Res Int. 2021 Jul 12;2021:8849415. doi: 10.1155/2021/8849415 (PMC8292096; doi:10.1155/2021/8849415)
Supplement: Supplementary Materials — See Table S1 for the connection between clinicopathological parameters and expression levels of ten hub genes. [file 8849415.f1.pdf]

Table S1. Association between mRNA expression difference of each gene and clinicopathological parameters in HCC

Parameters	CYP3A4			UGT1A6			AOX1			UGT1A4			UGT2B15		
	n	Mean±SD	P	n	Mean±SD	P	n	Mean±SD	P	n	Mean±SD	P	n	Mean±SD	P
**Gender**															
Male	46	-8.407±4.404	0.605	46	-0.754±3.488	0.824	46	-6.212±3.216	0.361	45	-5.046±3.857	0.775	46	-3.558±3.448	0.545
Female	13	-9.382±6.236		13	-1.007±3.965		13	-7.533±4.757		13	-5.493±5.122		13	-4.448±4.852	
**Age(years)**															
>60	30	-7.991±5.182	0.311	30	-0.804±3.858	0.990	30	-6.304±4.023	0.669	29	-4.844±4.295	0.581	30	-3.813±4.406	0.904
<=60	29	-9.274±4.416		29	-0.816±3.300		29	-6.710±3.178		29	-5.449±4.007		29	-3.693±3.061	
**Smoking**															
Yes	22	-8.308±4.290	0.703	22	-0.461±3.482	0.567	22	-6.178±3.258	0.597	22	-5.372±4.367	0.748	22	-3.435±3.086	0.620
No	37	-8.808±5.162		37	-1.017±3.643		37	-6.697±3.830		36	-5.009±4.032		37	-3.944±4.156	
**Drinking**															
Yes	21	-8.278±4.146	0.688	21	-0.578±3.689	0.713	21	-5.801±3.351	0.270	21	-5.218±4.464	0.922	21	-3.066±2.868	0.302
No	38	-8.812±5.203		38	-0.938±3.536		38	-6.892±3.727		37	-5.106±3.988		38	-4.135±4.177	
**Family history**															
Positive	7	-10.361±4.580	0.314	7	0.847±2.788	0.193	7	-7.837±3.778	0.302	7	-4.843±4.297	0.838	7	-3.285±3.664	0.729
Negative	52	-8.388±4.849		52	-1.033±3.620		52	-6.324±3.583		51	-5.188±4.147		52	-3.818±3.817	
**Body Mass Index(kg/m^2)**															
>=27.5	4	-6.737±6.256	0.414	4	-3.694±3.526	0.101	4	-6.990±5.566	0.821	4	-6.245±6.285	0.617	4	-3.017±2.616	0.674
<27.5	54	-8.810±4.775		54	-0.693±3.470		54	-6.567±3.453		53	-5.170±3.961		54	-3.855±3.876	
**Maximum tumor size(cm)**															
>5	30	-8.970±4.830	0.479	30	-1.171±3.895	0.308	30	-6.598±3.871	0.644	30	-5.643±4.324	0.347	30	-4.471±4.022	0.119
<=5	28	-8.065±4.844		28	-0.222±3.049		28	-6.164±3.174		28	-4.614±3.914		28	-2.912±3.425	
**Histological differentiation**															
poor differentiated	13	-11.488±3.890	**0.007**	13	-1.596±3.462	0.188	13	-8.498±2.247	**0.003**	13	-7.541±3.929	**0.006**	13	-4.057±3.626	0.565
well to moderate	41	-7.520±4.563		41	-0.199±3.238		41	-5.453±3.288		41	-4.182±3.630		41	-3.397±3.564	
**Tumor necrosis**															
Positive	23	-10.075±4.620	0.064	23	-1.054±4.140	0.678	23	-6.730±2.910	0.703	23	-5.769±4.072	0.356	23	-5.424±3.972	**0.006**
Negative	36	-7.693±4.781		36	-0.654±3.195		36	-6.359±4.023		35	-4.737±4.171		36	-2.688±3.266	
**Satellite lesions**															
Positive	19	-10.259±4.709	0.072	19	-1.860±4.188	0.120	19	-7.991±3.746	**0.028**	19	-7.035±4.500	**0.014**	19	-5.582±4.627	**0.029**
Negative	40	-7.844±4.736		40	-0.311±3.162		40	-5.797±3.359		39	-4.226±3.649		40	-2.886±2.981	
**Vascular cancer embolus**															
Positive	18	-10.070±3.961	0.127	18	-0.267±3.363	0.442	18	-7.715±2.599	**0.049**	18	-5.804±4.064	0.420	18	-4.650±3.475	0.230
Negative	41	-7.986±5.069		41	-1.049±3.663		41	-5.972±3.879		40	-4.851±4.173		41	-3.361±3.871	
**pT**															
T3-4	24	-9.634±4.703	0.184	24	-1.657±4.077	0.132	24	-7.158±3.534	0.252	24	-6.109±4.397	0.137	24	-4.774±4.333	0.085
T0-2	35	-7.928±4.847		35	-0.229±3.093		35	-6.055±3.639		34	-4.467±3.848		35	-3.055±3.216	
**pN**															
N1	5	-13.176±2.028	**0.002**	5	-3.414±3.201	0.088	5	-8.918±1.904	0.119	5	-8.653±2.997	**0.046**	5	-7.185±1.682	**0.032**
N0	54	-8.200±4.796		54	-0.569±3.525		54	-6.280±3.656		53	-4.816±4.086		54	-3.437±3.761	
**Cirrhosis**															
Yes	35	-8.036±4.326	0.264	35	0.293±2.827	**0.006**	35	-5.725±3.107	0.057	35	-4.252±3.749	**0.041**	35	-3.333±3.417	0.304
No	24	-9.475±5.451		24	-2.419±3.958		24	-7.639±4.034		23	-6.508±4.383		24	-4.369±4.239	
**HBsAg**															
Present	40	-8.565±4.468	0.897	40	-0.351±3.192	0.153	40	-6.436±3.275	0.836	40	-5.201±4.147	0.882	40	-3.634±3.316	0.725
Absent	19	-8.742±5.628		19	-1.777±4.171		19	-6.647±4.319		18	-5.024±4.202		19	-4.009±4.684	
**Serum AFP (μg/L)**															
>20	22	-8.460±4.412	0.836	22	0.341±2.794	**0.032**	22	-6.106±3.481	0.543	22	-4.795±3.628	0.576	22	-3.378±3.689	0.548
<=20	36	-8.737±5.179		36	-1.582±3.839		36	-6.712±3.756		35	-5.434±4.485		36	-4.004±3.906	
**Serum CEA (μg/L)**															
>5	7	-8.898±6.038	0.879	7	-0.658±3.694	0.879	7	-6.670±4.546	0.886	7	-5.874±4.308	0.645	7	-2.438±5.208	0.482
<=5	51	-8.595±4.753		51	-0.879±3.601		51	-6.457±3.546		50	-5.092±4.166		51	-3.949±3.600	
**Serum CA19-9 (U/mL)**															
>37	19	-9.436±4.732	0.384	19	-1.243±3.671	0.567	19	-6.644±3.480	0.816	18	-5.486±4.341	0.716	19	-4.083±3.444	0.662
<=37	39	-8.240±4.940		39	-0.663±3.567		39	-6.404±3.750		39	-5.050±4.113		39	-3.613±4.002	
**Serum CA125 (U/mL)**															
>35	12	-9.584±4.909	0.436	12	-1.279±2.568	0.323	12	-7.618±3.640	0.239	12	-6.100±3.862	0.318	12	-5.246±3.486	0.089
<=35	31	-8.343±4.542		31	-0.147±3.564		31	-6.201±3.428		31	-4.773±3.857		31	-3.082±3.720	
**Serum TB (μmol/L)**															
>24	8	-10.448±3.973	0.253	8	-1.946±3.938	0.336	8	-7.576±3.604	0.370	7	-5.477±4.501	0.824	8	-3.407±3.201	0.782
<=24	51	-8.335±4.915		51	-0.632±3.510		51	-6.335±3.614		51	-5.101±4.120		51	-3.809±3.880	
**Serum ALT (U/L)**															
>40	17	-8.775±4.570	0.878	17	-1.143±3.859	0.652	17	-6.099±3.771	0.588	16	-5.329±4.846	0.838	17	-3.420±2.729	0.669
<=40	42	-8.560±4.974		42	-0.675±3.477		42	-6.667±3.572		42	-5.077±3.883		42	-3.890±4.143	
**Serum albumin (g/L)**															
<40	36	-9.594±4.632	0.052	36	-1.194±3.744	0.305	36	-6.847±3.474	0.364	35	-5.487±4.039	0.444	36	-4.419±4.017	0.091
>=40	23	-7.101±4.820		23	-0.210±3.250		23	-5.965±3.822		23	-4.629±4.298		23	-2.714±3.161	
**Plasma PT(s)**															
>13.6	11	-10.030±4.205	0.287	11	-0.900±3.501	0.927	11	-7.156±2.811	0.510	11	-5.168±3.724	0.985	11	-4.798±4.307	0.313
<=13.6	48	-8.299±4.937		48	-0.789±3.614		48	-6.354±3.775		47	-5.141±4.254		48	-3.515±3.647	

Parameters	CDK1			CCNB1			MAD2L1			CCNB2			CDC20		
	n	Mean±SD	P	n	Mean±SD	P	n	Mean±SD	P	n	Mean±SD	P	n	Mean±SD	P
**Gender**															
Male	45	2.676±2.017	0.609	45	1.734±1.866	0.917	46	-0.986±1.761	0.142	46	2.939±1.818	0.723	45	0.842±2.144	0.638
Female	13	2.336±2.356		12	1.802±2.421		13	-0.104±2.297		13	3.146±1.957		11	0.499±2.191	
**Age(years)**															
>60	30	2.896±2.114	0.265	30	1.997±1.898	0.320	30	-0.627±1.722	0.505	30	3.241±1.678	0.279	29	0.811±1.912	0.898
<=60	28	2.282±2.034		27	1.472±2.050		29	-0.961±2.097		29	2.720±1.977		27	0.736±2.393	
**Smoking**															
Yes	21	2.791±1.827	0.602	22	2.007±1.490	0.437	22	-1.104±1.404	0.286	22	3.262±1.621	0.375	22	1.046±1.960	0.450
No	37	2.491±2.228		35	1.586±2.227		37	-0.605±2.147		37	2.820±1.953		34	0.599±2.256	
**Drinking**															
Yes	20	2.812±1.935	0.577	21	1.700±1.852	0.889	21	-1.140±1.462	0.301	21	3.066±1.757	0.803	21	1.022±2.087	0.508
No	38	2.488±2.170		36	1.777±2.063		38	-0.599±2.106		38	2.940±1.898		35	0.627±2.184	
**Family history**															
Positive	7	2.742±1.660	0.849	6	1.495±1.265	0.743	7	-0.644±2.328	0.829	7	2.727±1.732	0.696	6	0.289±1.967	0.561
Negative	51	2.580±2.145		51	1.778±2.045		52	-0.811±1.869		52	3.019±1.861		50	0.833±2.168	
**Body Mass Index(kg/m^2)**															
>=27.5	4	1.217±3.134	0.170	4	0.421±2.088	0.166	4	-1.781±2.091	0.283	4	1.957±2.933	0.237	4	-0.339±2.761	0.278
<27.5	53	2.717±2.002		52	1.857±1.964		54	-0.703±1.907		54	3.091±1.748		51	0.883±2.106	
**Maximum tumor size(cm)**															
>5	29	2.561±2.119	1.000	29	1.671±2.035	0.799	30	-0.924±1.899	0.672	30	2.949±1.760	0.977	29	0.888±2.193	0.630
<=5	28	2.561±2.075		27	1.809±1.971		28	-0.709±1.949		28	2.935±1.916		26	0.605±2.137	
**Histological differentiation**															
poor differentiated	13	3.606±1.832	**0.036**	12	1.891±1.788	0.659	13	-0.119±1.785	0.092	13	3.647±1.753	0.099	12	1.248±1.835	0.265
well to moderate	40	2.321±1.880		40	1.611±1.954		41	-1.115±1.838		41	2.698±1.784		39	0.507±2.037	
**Tumor necrosis**															
Positive	23	2.628±1.928	0.935	22	1.827±1.565	0.800	23	-0.702±1.756	0.777	23	3.030±1.612	0.881	22	0.960±1.953	0.606
Negative	35	2.581±2.203		35	1.699±2.209		36	-0.848±2.018		36	2.956±1.985		34	0.655±2.269	
**Satellite lesions**															
Positive	19	3.160±2.120	0.154	18	2.303±1.834	0.151	19	-0.298±1.689	0.173	19	3.585±1.387	0.083	18	1.486±2.146	0.087
Negative	39	2.326±2.032		39	1.493±2.002		40	-1.026±1.978		40	2.700±1.964		38	0.438±2.077	
**Vascular cancer embolus**															
Positive	18	2.440±1.998	0.698	16	2.076±2.288	0.438	18	-0.191±2.355	0.110	18	3.222±1.904	0.514	15	0.746±2.380	0.952
Negative	40	2.671±2.138		41	1.621±1.849		41	-1.055±1.637		41	2.880±1.817		41	0.785±2.073	
**pT**															
T3-4	24	2.920±2.225	0.329	23	1.986±1.972	0.459	24	-0.402±1.859	0.196	24	3.176±1.890	0.512	23	1.114±2.252	0.327
T0-2	34	2.374±1.975		34	1.588±1.984		35	-1.058±1.919		35	2.854±1.811		33	0.539±2.055	
**pN**															
N1	5	2.501±1.078	0.913	5	0.872±1.000	0.302	5	-1.410±1.004	0.453	5	2.608±1.277	0.635	5	0.182±1.041	0.277
N0	53	2.609±2.158		52	1.833±2.026		54	-0.734±1.965		54	3.020±1.883		51	0.833±2.214	
**Cirrhosis**															
Yes	34	2.661±1.958	0.791	34	2.232±1.929	**0.023**	35	-0.674±1.857	0.573	35	3.157±1.645	0.389	33	1.215±2.142	0.064
No	24	2.512±2.283		23	1.034±1.848		24	-0.962±2.004		24	2.734±2.091		23	0.143±2.009	
**HBsAg**															
Present	39	2.521±1.868	0.686	39	1.915±1.895	0.352	40	-0.901±1.763	0.527	40	3.049±1.675	0.697	38	0.966±2.093	0.336
Absent	19	2.760±2.510		18	1.387±2.139		19	-0.561±2.212		19	2.848±2.176		18	0.372±2.235	
**Serum AFP (μg/L)**															
>20	21	3.025±1.987	0.242	21	2.139±2.190	0.190	22	-0.705±1.979	0.723	22	3.176±1.753	0.507	21	1.399±2.232	**0.050**
<=20	36	2.346±2.149		35	1.430±1.768		36	-0.891±1.891		36	2.840±1.913		34	0.271±1.895	
**Serum CEA (μg/L)**															
>5	7	4.265±2.005	**0.023**	7	2.794±2.372	0.112	7	0.214±1.518	0.128	7	4.019±1.596	0.109	7	2.083±1.966	0.060
<=5	50	2.362±2.022		49	1.539±1.857		51	-0.962±1.926		51	2.823±1.844		48	0.500±2.043	
**Serum CA19-9 (U/mL)**															
>37	19	3.148±1.798	0.162	18	1.901±1.538	0.592	19	-0.367±1.635	0.210	19	3.368±1.461	0.252	18	0.974±1.788	0.504
<=37	38	2.320±2.205		38	1.599±2.128		39	-1.041±2.013		39	2.772±1.995		37	0.569±2.226	
**Serum CA125 (U/mL)**															
>35	11	2.120±1.835	0.355	12	1.762±2.021	0.907	12	-1.051±1.669	0.627	12	2.865±1.609	0.681	12	1.089±2.577	0.540
<=35	31	2.796±2.123		30	1.844±2.066		31	-0.727±2.043		31	3.115±1.842		29	0.634±1.947	
**Serum TB (μmol/L)**															
>24	8	3.239±2.254	0.354	7	1.702±2.009	0.947	8	-0.166±2.139	0.322	8	3.287±1.963	0.620	7	0.781±1.982	0.993
<=24	50	2.497±2.057		50	1.755±1.987		51	-0.889±1.871		51	2.937±1.829		49	0.774±2.178	
**Serum ALT (U/L)**															
>40	16	2.636±1.986	0.935	16	1.416±1.881	0.431	17	-1.041±1.989	0.527	17	2.609±1.847	0.321	16	0.740±2.079	0.939
<=40	42	2.586±2.139		41	1.878±2.013		42	-0.690±1.887		42	3.137±1.829		40	0.789±2.186	
**Serum albumin (g/L)**															
<40	35	2.882±2.016	0.206	36	2.242±1.818	**0.012**	36	-0.417±1.596	0.058	36	3.464±1.643	**0.011**	35	1.264±2.080	**0.026**
>=40	23	2.170±2.149		21	0.902±1.976		23	-1.378±2.222		23	2.235±1.900		21	-0.041±2.022	
**Plasma PT(s)**															
>13.6	11	3.328±1.706	0.200	10	2.320±1.860	0.317	11	-0.285±1.821	0.333	11	3.284±1.821	0.553	10	1.451±1.808	0.274
<=13.6	47	2.429±2.139		47	1.627±1.992		48	-0.907±1.925		48	2.916±1.850		46	0.628±2.192	

HCC hepatocellular carcinoma; HBsAg hepatitis B surface antigen; AFP alpha-fetoprotein; CEA carcinoembryonic antigen; CA19-9 carbohydrate antigen 19-9; CA125 carbohydrate antigen 125; TB total bilirubin; ALT alanine aminotransferase; PT prothrombin time; P value：independent-sample t test.
